# Therapeutic strategies for preventing skeletal muscle fibrosis after injury

**DOI:** 10.3389/fphar.2015.00087

**Published:** 2015-04-21

**Authors:** Koyal Garg, Benjamin T. Corona, Thomas J. Walters

**Affiliations:** US Army Institute of Surgical Research, Extremity Trauma and Regenerative MedicineHouston, TX, USA

**Keywords:** TGF-ß1, fibrosis, muscle injury, muscle regeneration, extracellular matrix

## Abstract

Skeletal muscle repair after injury includes a complex and well-coordinated regenerative response. However, fibrosis often manifests, leading to aberrant regeneration and incomplete functional recovery. Research efforts have focused on the use of anti-fibrotic agents aimed at reducing the fibrotic response and improving functional recovery. While there are a number of mediators involved in the development of post-injury fibrosis, TGF-β1 is the primary pro-fibrogenic growth factor and several agents that inactivate TGF-β1 signaling cascade have emerged as promising anti-fibrotic therapies. A number of these agents are FDA approved for other conditions, clearing the way for rapid translation into clinical treatment. In this article, we provide an overview of muscle's host response to injury with special emphasis on the cellular and non-cellular mediators involved in the development of fibrosis. This article also reviews the findings of several pre-clinical studies that have utilized anti-fibrotic agents to improve muscle healing following most common forms of muscle injuries. Although some studies have shown positive results with anti-fibrotic treatment, others have indicated adverse outcomes. Some concerns and questions regarding the clinical potential of these anti-fibrotic agents have also been presented.

## Introduction

According to the Armed Forces Health Surveillance Center, musculoskeletal injuries were the leading cause of medical encounters in 2010 (A.F.H.S. Center, 2011; Zambraski and Yancosek, 2012). On the battlefield, musculoskeletal trauma constitutes the majority of injuries (Owens et al., 2008). In civilian medicine muscle injuries related to sports account for 10–55% of all injuries (Garrett, 1996; Huard et al., 2002; Jarvinen et al., 2005). The poor healing responses and a high risk of re-injury presents a significant challenge to the performance of a service member or a professional athlete. The inability to effectively treat these injuries can have devastating consequences including permanent functional deficits, failed limb salvage, and delayed amputation, resulting in a tremendous toll on quality of life for the wounded personnel and their families, and also represents a great expense to the military in terms of military readiness and medical costs.

Muscle trauma can range from simple strains and contusions to severe lacerations and penetrating trauma, including volumetric muscle loss (VML) (physical loss of muscle). The ability of the skeletal muscle to regenerate depends on the type and severity of the injury. While skeletal muscle has a remarkable regenerative capacity even simple strains can heal incompletely resulting in vulnerability to reinjury (Carlson, 1986; Huard et al., 2002; Mu et al., 2010). Severe battlefield trauma involving VML is well beyond the muscles inherent capacity for self-repair (Grogan and Hsu, 2011).

The major impediment to optimal muscle healing after any injury is fibrosis (Huard et al., 2002), defined as an abnormal and unresolvable, chronic overproliferation of extracellular matrix (ECM) components (Lieber and Ward, 2013). Fibrosis interferes with muscle regeneration (Huard et al., 2002), causes a loss in muscle function (Lieber and Ward, 2013) and alters the tissue environment causing increased susceptibility to reinjury (Carlson, 1986; Huard et al., 2002; Mu et al., 2010).

Clearly treatments aimed at improving muscle healing following injury would be of great benefit. Efforts in this area have concentrated on enhancing muscle regeneration and reducing fibrosis. Of the two, the greatest effort has been devoted to enhancing regeneration, primarily related to growth factors and/or cell-based treatments. Although optimal healing clearly involves both processes, this review will focus on therapies aimed at improving healing specifically through the reduction of fibrosis.

## Fibrosis: key players and contributing factors

### Extracellular matrix of skeletal muscle

The muscle ECM is composed of two major layers; the basal lamina and the interstitial matrix. The basal lamina is in direct contact with the sarcolemma, and is composed primarily of type IV collagen, laminin, and heparan sulfate proteoglycans. The more abundant interstitial matrix surrounds the basal lamina and is primarily composed of collagen I, III, and V, fibronectin and perlecan (Cornelison, 2008). Structurally the ECM provides mechanical support, organization and directional guidance for nerves, vessels, and muscle cells (Sanes, 2003; Cornelison, 2008). It also provides the overall anatomical organization of the muscle: the endomysium surrounds each individual myofiber; the perimysium surrounds groups of myofibers to form fascicles; and epimysium surrounds each muscle. The perimysium contains primarily collagen I, whereas type III collagen is evenly distributed between endomysium and epimysium (Light and Champion, 1984; Gillies and Lieber, 2011).

Functionally, the ECM plays multiple roles. It is the primary contributor to the passive elastic properties of the muscle. Alterations in the amount or composition of collagen as a result of injury, diseases or aging is reflected as alterations in muscle stiffness (Lieber and Ward, 2013). The ECM and muscle fiber are coupled through intricate protein networks composed of dystroglycan and α/β integrin complexes that connect both the contractile proteins and the nucleus within the interior of the muscle fiber to the sarcolemma membrane and in turn to the basal lamina of the ECM (Jaalouk and Lammerding, 2009). These connections provide a means to transmit force from the contractile elements of individual muscle fibers to the ECM, which are in turn transmitted to the tendon and ultimately the bone (Kjaer et al., 2006). It also provides a means to transmit force laterally through providing a connection among individual neighboring muscle fibers, as well as among fascicles (Street and Ramsey, 1965; Kjaer, 2004). The protein complexes also facilitate the transduction of mechanical cues to the nucleus of the muscle and for the presentation of sequestered growth factors such as hepatocyte growth factor (HGF) and fibroblast growth factor (FGF) to the muscle (Cornelison, 2008). This cross-talk provides the requisite communication to tune the needs of the muscle to its mechanical environment for proper cell differentiation during development (Reilly and Engler, 2010) and repair (Kjaer et al., 2006), as well as for adaptation to altered physical demands (Kjaer et al., 2006).

### Normal vs. aberrant regeneration of skeletal muscle after injury

The host response to muscle injury consists of three broad phases: degeneration (1–3 days), regeneration (3–4 weeks) and remodeling (3–6 months) (Jarvinen et al., 2005; Smith et al., 2008). The degeneration phase is characterized by the disruption of muscle ultrastructure and ensuing necrosis of the damaged muscle fibers. Entry of plasma proteins and activation of the complement cascade induces chemotactic recruitment of inflammatory cells (Tidball, 2005). Plasma proteins such as fibrin cross-link and invading fibroblasts deposit collagen to form a provisional ECM matrix. This ECM matrix is transient and acts as a scaffold for the invading cells and supports the ruptured and damaged myofibers during the ongoing healing process (Middleton and Smith, 2007; Smith et al., 2008). Neutrophils are typically the first immune responders, which are gradually replaced by macrophages as the predominant inflammatory cell at the site of injury (Tidball, 2005; Tidball and Villalta, 2010). The duration and intensity of the inflammatory response after muscle injury can critically influence the regeneration process. Macrophages begin the disinfection and debridement of the wound site by phagocytosis of necrotic muscle fibers, cellular debris and microorganisms (Wynn, 2004; Smith et al., 2008; Tidball and Villalta, 2010; Wehling-Henricks et al., 2010; Wynn and Barron, 2010). This macrophage population is classified as the M1 phenotype and is pro-inflammatory. Cytokines released by the M1 macrophages (e.g., Tumor necrosis factor - alpha, interleukin (IL)-6) promote recruitment, activation and proliferation of muscle satellite cells (Torrente et al., 2003; Lolmede et al., 2009), the primary muscle precursor. The regenerative phase ensues with satellite cell proliferation, which leads to the formation of myogenic precursor cells called myoblasts which express myogenic transcription factors such as MyoD and Myf5 (Yan et al., 2003). For proper muscle healing, a shift in the macrophage phenotype from a pro-inflammatory M1 to a tissue remodeling M2 is absolutely essential (Arnold et al., 2007). M2 macrophages peak in numbers at ~4 days post injury and persist until the muscle remodeling phase (Tidball, 2005; Tidball and Villalta, 2010). M2s promote myoblast differentiation and fusion and maturation of myotubes by releasing IL-4 and IL-10. At this time the expression of myogenin, Myf4 and myocyte enhancer binding factor-2 (MEF2) is initiated (Lluis et al., 2006; Le Grand and Rudnicki, 2007; Rudnicki et al., 2008). The newly forming myotubes fuse with the existing myofibers to form new muscle tissue mature muscle fibers. The regenerative phase overlaps with the remodeling phase, in which maturation of the regenerating fibers into a functional contractile unit takes place. In the final phases of remodeling, re-organization of the ECM, revascularization and reinnervation of the myofibers occurs to ensure structural and functional recovery (Ciciliot and Schiaffino, 2010).

### The development of fibrosis

ECM deposition in the wound bed can be seen within a week post-injury and it can continue on for several weeks. The predominant cell type responsible for the deposition of ECM is the fibroblast. In response to locally produced mediators such as transforming growth factor beta 1 (TGF-β1), fibroblasts transform into α-smooth muscle actin (α-SMA) expressing myofibroblasts (Darby et al., 1990; Desmouliere et al., 2005). These cells play a key role in wound healing and matrix deposition. Myofibroblasts can also arise from endothelial or epithelial cells or from epithelial stem cell progenitors via endothelial-mesenchymal transition. In addition, circulating CD34^+^ bone marrow derived progenitor cells called fibrocytes can also be recruited to the wound site to promote collagen deposition (Quan et al., 2004). Among the first synthesized ECM proteins by the myofibroblasts in the wound bed are fibronectin and tenascin-C (Hanamura et al., 1997; Tuxhorn et al., 2002), followed by collagen type III and collagen type I. As the production of collagen type I continues on for several weeks, the tensile strength of the scar tissue increases considerably (Kaariainen et al., 1998). In cases of acute and self-healing injuries (e.g., muscle strains), myofibroblasts disappear after wound closure due to apoptotic signals. But in cases of chronic injuries marked by persistent inflammation (e.g., VML), these cells do not undergo apoptosis and remain in the granulation tissue. The sustained presence and elevated numbers of immune cells in the granulation tissue promote the release of fibrogenic cytokines such as TGF-β1. Under these conditions, myofibroblasts continue to proliferate and synthesize ECM, thus contributing to pathological scar tissue formation, referred to as fibrosis (Desmoulière et al., 2003; Moulin et al., 2004; Sarrazy et al., 2011).

ECM deposition typically proceeds more rapidly than myogenesis. If unresolved under physiological conditions, this ECM transforms into a fibrotic scar that creates a mechanical barrier and restricts the regeneration of myofibers and axons across the injury gap (Jarvinen and Lehto, 1993; Jarvinen et al., 2005, 2007). Furthermore, the fibrotic tissue lacks the elasticity of the native muscle, which can render the muscle susceptible to reinjury (Huard et al., 2002).

### Role of TGF-β1 and factors in fibrosis and regeneration

**Figure 1:**
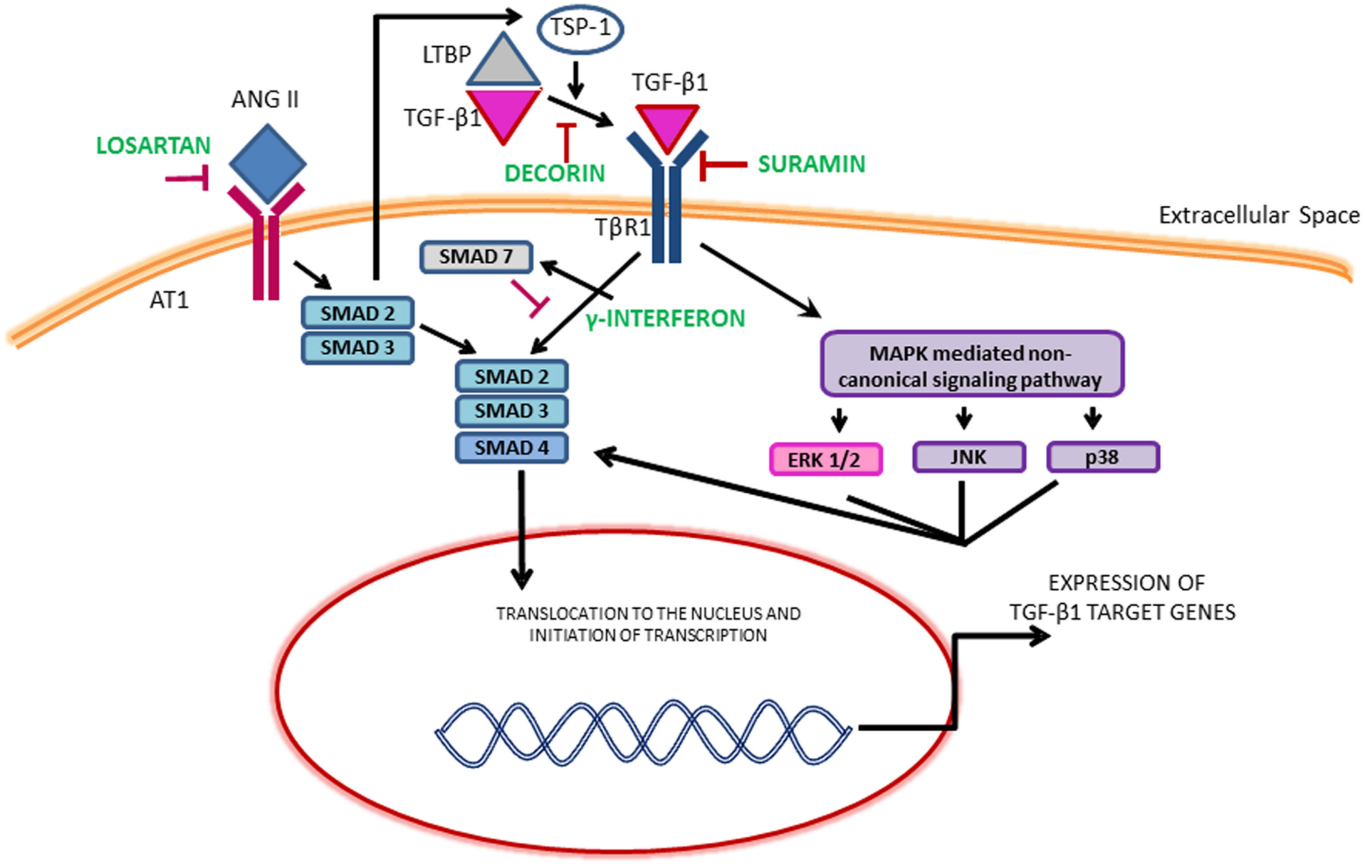
**Illustration of the TGF-β1 signaling pathways and the mechanism of therapeutics**. ERK, Extracellular signal regulated kinase; JNK, c-Jun N-terminal kinase; LTBP, Latent transforming growth factor binding proteins; MAPKs, Mitogen-activated protein kinase; TSP-1, Thrombospondin-1.

Although several growth factors such as epidermal growth factor (EGF), vascular endothelial growth factor (VEGF) and FGF-2 released from neutrophils, macrophages, fibroblasts and myogenic precursors can promote fibrosis, the most pro-fibrogenic growth factor identified in the literature is TGF-β1 (Sheppard, 2006; Serrano and Munoz-Canoves, 2010; Burks and Cohn, 2011; Mann et al., 2011; Serrano et al., 2011). In the canonical TGF-β1 pathway, ligand binding leads to the phosphorylation of SMAD2 and SMAD3 which then bind to a common mediator SMAD4 and translocate to the nucleus to activate collagen transcription. SMAD7 suppresses this action. TGF-β1 can also signal through the induction of non-canonical pathways including mitogen activated protein kinase (MAPK). The MAPK family consists of isoforms of extracellular signal-regulated kinases (ERKs), c-Jun N-terminal kinase (JNKs) and p38 (Figure 1). The activation of MAPK pathway may also phosphorylate SMADs independent of the canonical TGF-β1 pathway. Both these pathways lead to the synthesis of ECM proteins, cell proliferation, differentiation and motility. The effects of TGF-β1 can also be mimicked and amplified by other growth factors and members of the TGF-β1 superfamily such as connective tissue growth factor (CTGF), myostatin and platelet derived growth factor (PDGF-AA, BB) (Sheppard, 2006; Pohlers et al., 2009).

The maintenance of the ECM involves a delicate equilibrium between MMPs and their inhibitors; tissue inhibitors of metalloproteinases (TIMPs). Matrix metalloproteinases (MMPs) are endogenous zinc-dependent proteases capable of degrading ECM components. TIMPs inhibit the enzymatic activity of MMPs either by binding to the active MMPs or stabilizing the inactive forms. MMPs expressed in skeletal muscle include MMP-1, 2, 9, and 13. MMP-2 and MMP-9 are gelatinases that degrade type IV collagen, fibronectin, proteoglycans and laminin. MMP-1 and MMP-13 degrade type I and III collagen. Besides matrix destruction, MMPs also play other crucial roles in cell-to-cell communication and myogenesis (Kherif et al., 1999; Chen and Li, 2009; Gillies and Lieber, 2011).

TGF-β1 can promote fibrosis through aberrant ECM deposition and decreased production of MMPs, thereby promoting the survival of myofibroblasts and preventing the destruction of the deposited ECM (Vaday et al., 2001; Yuan and Varga, 2001; Serrano et al., 2011). Additionally, TGF-β1 inhibits satellite cell and myoblast proliferation and differentiation (Allen and Boxhorn, 1989; Johnson and Allen, 1990; Li et al., 2008). TGF-β1 can also promote fibrotic cascades via the differentiation of myoblasts and muscle derived stem cells into myofibroblasts (Li et al., 2004). TGF-β1 has anti-inflammatory functions and can switch M1 macrophages to an M2 phenotype. Macrophages of the M2 phenotype produce various growth factors such as TGF-β1, PDGF, FGF-2, and VEGF and express high levels of arginase, which can all potentially lead to increased matrix production (Wehling-Henricks et al., 2010). In contradiction, other studies have suggested that M2s are required for the suppression and resolution of fibrosis because they can also stimulate the production of collagen degrading MMPs and IL-10 (Pesce et al., 2009; Wynn and Ramalingam, 2012). A recent study showed that exogenous therapy of M1 macrophages reduced fibrosis and enhanced muscle fiber regeneration in lacerated muscles (Novak et al., 2014). Thus, macrophages play very complex roles in regeneration and are capable of both inducing and inhibiting fibrosis (Wynn, 2004; Wynn and Barron, 2010; Murray and Wynn, 2011).

## Anti-fibrotic therapies

The dominant role of TGF-β1 makes it an obvious target for anti-fibrotic treatments and several agents that inactivate TGF-β1 signaling cascade have emerged as promising therapies. Table 1 provides an overview of the existing agents in the literature. The focus of this review article is on muscle injury. Therefore, the readers are referred elsewhere for information on anti-fibrotic agents aimed at muscle diseases (e.g., pirfenidone) (Burks and Cohn, 2011). Losartan is an FDA approved antihypertensive medication that acts by blocking angiotensin II type 1 receptor (AT1) (Figure 1). Activation of the AT1 receptor by angiotensin produces thrombospondin -1 (TSP-1), which is a key regulator of latent TGF-β1 activation. Losartan is an inhibitor of AT1 activation and indirectly blocks TGF-β1 activation by inhibiting TSP-1 production (Chamberlain, 2007). Losartan has been shown to attenuate TGF-β1 signaling in chronic renal disease, cardiomyopathies and marfan syndrome (Cohn et al., 2007). The optimal healing dose of losartan for muscle healing coincides with the clinically relevant safe human dose of 10 mg/kg/day, which is easily administered in drinking water of rodents (Kobayashi et al., 2013).

**Table 1:** **Comprehensive summary of antifibrotic medications used for the treatment of muscle injury or disease**.

**Drug**	**Mechanism of action**	**Injury or disease model**	**Animal model**	**Administration**	**Results of the study**	**Side effects**	**Refferences**
**FDA APPROVED ANTIFIBROTIC MEDICATIONS**							
Losartan	Angiotensin 1 receptor antagonist	Muscle laceration injury	C57BL/6J mice	Oral (0.5 g/L) daily for 3-5 weeks	Decreased fibrosis and improved regeneration	Hypotension, headache, dizziness, fatigue, cholestatic hepatitis, raised liver enzymes, and pancreatitis	Bedair et al., 2008; Park et al., 2012
			C57BL/6-Tg	Oral (0.6 g/L) for 2 weeks	Improved regeneration and inhibition of fibrosis when combined with ASCs		
		Muscle contusion injury	C57BL/6J mice	Oral (10 mg/kg/day) from 3 and 7 days post-injury till sac	Decreased fibrosis and improved regeneration with functional recovery		Kobayashi et al., 2013; Terada et al., 2013
				Oral (10 mg/kg/day) from Day 3 post injury till sac	Decreased fibrosis, improved regeneration with functional recovery in combination with PRP		
		Volumetric muscle loss injury	Lewis rats	Oral (10 mg/kg/day) from Day 3 post injury till 28 days	Decreased fibrosis but hindered functional recovery		Garg et al., 2014
		Duchenne Muscular Dystrophy (DMD)	C57BL/10ScSn *mdx* mice	Oral (0.6 g/L) for 6 months	Improved skeletal and cardiac muscle regeneration and function		Spurney et al., 2011
		Marfan Syndrome	C57BL/6J Fbn^1C1039G/+^mice	Oral (0.6 g/L) for 6 months	Improved muscle regeneration and function		Cohn et al., 2007
Suramin	TGF-β1 receptor antagonist	Muscle contusion, laceration and strain injuries	C57BL/6J, C57BL/10J mice	Intramuscular injection (2.5 mg/20 μL of PBS) at 0, 7, 14 days or at 2 weeks	Decreased fibrosis, improved regeneration and functional recovery	Malaise, neuropathy, mineral corticoid insufficiency, corneal deposits, thrombocytopenia, neutropenia and renal failure	Chan et al., 2003, 2005; Nozaki et al., 2008
		DMD	C57BL/10J *mdx* mice	I.P. (60 mg/kg) on alternate days for 7 days	Decreased fibrosis in diaphragm and skeletal muscles		Taniguti et al., 2011
γ-interferon	Induces Smad7 expression	Muscle laceration injury	C57BL/10J mice	Injected into the lacerated area (250 U/10 μL of PBS) at 2 weeks	Decreased fibrosis, improved regeneration and functional recovery	Chills, fever, malaise, fatigue, anorexia, alopecia, depression, loss of libido, dry skin and mouth	Foster et al., 2003
Pirfenidone	TGF-β1 antagonist	DMD	C57BL/10ScSn *mdx* mice	Oral (500 mg/kg) daily for 28 days or 1.2 g/100 mL	Improved cardiac function but no significant changes in fibrosis	Nausea, fatigue, dizziness, rash	Van Erp et al., 2006; Gosselin et al., 2007; Jiang et al., 2012
**OTHER ANTIFIBROTIC AGENTS**							
Decorin	TGF-β1 ligand binder	Muscle laceration injury	C57BL/10J mice	I.P. (50 μg/20 μL of PBS) at 0, 5, 10 and 15 days after injury	Decreased fibrosis, improved regeneration and functional recovery	Unknown	Fukushima et al., 2001
		DMD	C57BL/10ScSn *mdx* mice	I.P. (0.98 mg/mL) for 7 days daily	Decreased fibrosis		Gosselin et al., 2004
Halofuginone	Reduces Smad3 expression	DMD	C57BL/6J *mdx* mice	I.P. (7.5 μg) for 8 weeks	Decreased fibrosis, improved function of the cardiac and skeletal muscle		Huebner et al., 2008; Roffe et al., 2010
		Neonatal brachial plexus injury	CD1 mice	I.P. (0.3 μg/g) 3 times a week for 4 weeks	Decreased biceps fibrosis but did not reduce contracture severity		Nikolaou et al., 2014

Losartan has been shown to reduce the fibrotic area, improve muscle regeneration and improve muscle function in murine models of contusion (Kobayashi et al., 2013) and laceration (Bedair et al., 2008). However, the timing of administration is critical. Beneficial effects occur when administration begins on day 3 or 7 post-injury. In contrast, immediate administration results in aberrant regeneration, likely attributable to disruption of the initial inflammatory response and the natural healing process of skeletal muscle (Kobayashi et al., 2013). The anti-fibrotic effect of losartan has also been combined with other regenerative therapies in quest to further improve skeletal muscle healing. Losartan treatment has been shown to significantly improve the myogenic potential of transplanted ASCs (Park et al., 2012). Losartan combined with platelet rich plasma (PRP) significantly reduced fibrosis and improved function in a mouse contusion model compared to PRP alone (Terada et al., 2013). Although PRP presents a promising regenerative therapy (Sanchez et al., 2014), some researchers have raised concerns about the PRP-derived TGF-β in fibrotic remodeling of injured muscle (Robi and Matjaz, 2014). Regardless, these initial studies suggest that optimal treatment of muscle must consider the interactions of fibrosis and muscle regeneration. Although losartan is well tolerated, the side-effects include hypotension, headache, dizziness, fatigue, cholestatic hepatitis, raised liver enzymes and pancreatitis (Aronson, 2009).

Suramin is FDA approved as an anti-parasitic and anti-neoplastic agent that can inhibit several growth factors including TGF-β1 by competitively binding to their receptors (Chan et al., 2003). Intramuscular injection of suramin after injury caused by contusion (Nozaki et al., 2008, 2012), laceration (Chan et al., 2003) and strain (Chan et al., 2005) reduces fibrosis and improves functional recovery (Chan et al., 2003; Nozaki et al., 2008). Additionally, suramin also inhibits myostatin expression (Chan et al., 2005). The side-effects associated with suramin use include malaise, neuropathy, mineral corticoid insufficiency, corneal deposits, occasional thrombocytopenia, neutropenia and renal failure (Chan et al., 2005).

Gamma interferon (γ-INF) has also been shown to disrupt TGF-β1 signaling by upregulating smad7 expression and is approved by the FDA to treat hepatic fibrosis (Foster et al., 2003). In a mouse laceration model, γ-INF was shown to decrease fibrosis and improve muscle strength. The side effects of this drug include chills, fever, malaise, fatigue, anorexia, alopecia, depression, loss of libido and dry skin and mouth (Friedlander et al., 1996).

The proteoglycan decorin can bind to TGF-β1, preventing association with its receptor (Li et al., 2004), and has anti-fibrotic effects in kidney, liver and lung (Dreher et al., 1990; Isaka et al., 1996; Giri et al., 1997). It reduces fibrosis, and enhances muscle regeneration and function following muscle laceration in mice (Fukushima et al., 2001). Decorin has also been used in combination with IGF-1 in an attempt to reduce fibrosis and also enhance muscle regeneration. In a murine muscle laceration model the combination had an additive effect histologically. However, the results were not translated to an improvement in muscle function (Sato et al., 2003). The anti-fibrotic halofuginone has been shown to reduce fibrosis by reducing SMAD 3 phosphorylation. In a model of neonatal brachial plexus injury, 0.3 μg/g of halofuginone administration three times a week for 4 weeks decreased biceps fibrosis but did not reduce contracture severity (Nikolaou et al., 2014).

Other approaches for reducing muscle fibrosis after injury include MMP treatment. It was found that administration of recombinant human MMP-1 at day 33 post-laceration was effective in reducing muscle fibrosis (Kaar et al., 2008). It has also been suggested that increase in the activity of MMP-3 and MMP-9 by osteoactivin (a type 1 glycoprotein expressed in myofibers) is useful for attenuating skeletal muscle fibrosis caused by denervation and distraction osteogenesis (Furochi et al., 2007; Tonogai et al., 2014).

## Concerns and future directions

Unlike fibrosis, the increased collagen deposition is a normal response to altered demand including submaximal aerobic exercise (Miller et al., 2005), strength and resistance training (Moore et al., 2005; Heinemeier et al., 2007), and stretching (Stauber et al., 1996). In these cases, increased collagen deposition is a positive adaptive response that protects the muscle from strain injury and provides a means to improve lateral force transmission (Stauber et al., 1996; Gillies and Lieber, 2011). The increase in collagen deposition following these activities is largely affected by increased TGF-β and many of the same signals that stimulate collagen production also orchestrate positive adaptation within the myofibrils under these conditions. What remains unexplored is the interaction of anti-fibrotic treatment with the response to muscular activity, e.g., physical therapy.

There is also evidence that increased collagen deposition can also be beneficial response to certain forms of muscle injury. Some studies have challenged the concept of preventing fibrosis by blocking TGF-β signaling following muscle injury. Lieber and co-workers have suggested that the development of skeletal muscle fibrosis in response to nesprin and desmin deletion is a compensatory mechanism that protects muscle fiber from injury due to excessive strains (Meyer and Lieber, 2012; Chapman et al., 2014).

Gumucio et al. have demonstrated that inhibition of TGF-β using a bio-neutralizing antibody initially improved force production following eccentric contraction injury, however, it ultimately led to long-term force deficit (Gumucio et al., 2013). Recent work from our laboratory involving VML injury, a particularly severe form of muscle injury in which a portion of the muscle has been frank lost, demonstrated that the formation of a fibrotic scar partially restores muscle function (Nikolaou et al., 2014). Furthermore, accelerating scar formation through surgical repair and transplantation of an acellular ECM (Chen and Walters, 2013; Corona et al., 2013) or through exercise (Aurora et al., 2014), is accompanied by an improvement in muscle function. Conversely, muscle function is dramatically reduced when collagen deposition is delayed or reduced by the administration of losartan (Garg et al., 2014). In the unique case of VML injury, the development of a fibrotic scar at the wound site provides a means to transmit force between intact areas of muscle by providing a physical bridge. Additionally, the presence of scar at the wound site shields the remaining muscle from increased loading secondary to VML (Corona et al., 2013). Clearly, VML represents an extreme form of muscle injury, however, this work underlines the need to consider the type and magnitude of the injury when exploring anti-fibrotic treatments. TGF-β1 is a multi-functional growth factor with roles in inflammation, immunomodulation, wound healing and fibrosis (Kulkarni et al., 1993; Andreetta et al., 2006). Therefore, it is required to evaluate the long-term effects of anti-fibrotic therapies targeting TGF-β1 on immunomodulation. Systemic and prolonged attenuation of TGF-β1 may also lead to massive multi-organ inflammation and autoimmunity (Andreetta et al., 2006). Similarly, anti-fibrotic therapies such as γ-INF and halofuginone act downstream on the SMADs (Figure 1). SMADs are involved in a variety of different pathways besides fibrosis and interfering with their action could lead to undesirable effects (Rodriguez-Vita et al., 2005; Goldstein et al., 2011).

### Clinical translation

The translation of animal studies involving anti-fibrotic agents for muscle injury into human studies and clinical trials has been extremely limited. In fact, with the exception of a single case report involving the treatment of a muscle strain injury with losartan (Gharaibeh et al., 2012), we are unaware of any other human studies involving the treatment of muscle injury with an anti-fibrotic treatment. Clinically, muscle is viewed as a regenerative tissue and patients presenting with muscle injuries often do not receive medical treatment beyond R.I.C.E and some form of anti-inflammatory medication (Gharaibeh et al., 2012). Anti-fibrotic agents hold promise as advance in the treatment of muscle injury, however their potential side effects and possible disruption of normal adaptive responses represent a valid concern. While life-threating fibrotic diseases such as idiopathic pulmonary fibrosis and muscular dystrophy may warrant the potential adverse side effects of anti-fibrotic drugs, it is not clear where along the continuum of muscle injury the risk becomes worth the potential reward.

## Conclusion

Skeletal muscle repair following injury includes a complex and well-coordinated regenerative response. However, fibrosis often manifest, leading to aberrant regeneration and incomplete functional recovery. In general, preclinical animal studies have demonstrated improvements in muscle injuries with anti-fibrotic treatments. However, there remain a number of unanswered questions that will need to be addressed in order to refine our understanding of anti-fibrotic treatments and before their clinical potential is realized. For example: What is the optimal time to initiate treatment? What types of muscle injuries are most amenable to anti-fibrotic treatment, e.g. strain injures vs. VML injury? And do anti-fibrotic drugs impact the normal adaptive response of muscle to increased activity, therefore hindering long-term healing? While a number of potential treatments are FDA approved for other indications, clinical trials in human volunteers will be important in addressing concerns regarding potential side effects, particularly in regard to balancing cost vs. benefit of anti-fibrotic treatments. Finally, although anti-fibrotic treatments improve muscle healing in the majority of the studies reviewed, they do not result in complete muscle regeneration. To this end, recent studies combining anti-fibrotic treatments with cell-based therapies have provided exciting evidence that the optimal treatment of muscle injuries may lay in a multifactorial approach to treating muscle injuries.

### Conflict of interest statement

The authors declare that the research was conducted in the absence of any commercial or financial relationships that could be construed as a potential conflict of interest.
